# Mdm2 and MdmX RING Domains Play Distinct Roles in the Regulation of p53 Responses: A Comparative Study of Mdm2 and MdmX RING Domains in U2OS Cells

**DOI:** 10.3390/ijms21041309

**Published:** 2020-02-15

**Authors:** Olga Egorova, Heather HC Lau, Kate McGraphery, Yi Sheng

**Affiliations:** Department of Biology, York University, 4700 Keele Street, Toronto, ON M3J 1P3, Canada; olga-egorova@hotmail.com (O.E.); heather.lau@mail.utoronto.ca (H.H.L.); kate.mcgraphery@gmail.com (K.M.)

**Keywords:** Mdm2, MdmX, RING domain, the ubiquitin proteolytic system

## Abstract

Dysfunction of the tumor suppressor p53 occurs in most human cancers. Mdm2 and MdmX are homologous proteins from the Mdm (Murine Double Minute) protein family, which play a critical role in p53 inactivation and degradation. The two proteins interact with one another via the intrinsic RING (Really Interesting New Gene) domains to achieve the negative regulation of p53. The downregulation of p53 is accomplished by Mdm2-mediated p53 ubiquitination and proteasomal degradation through the ubiquitin proteolytic system and by Mdm2 and MdmX mediated inhibition of p53 transactivation. To investigate the role of the RING domain of Mdm2 and MdmX, an analysis of the distinct functionalities of individual RING domains of the Mdm proteins on p53 regulation was conducted in human osteosarcoma (U2OS) cell line. Mdm2 RING domain was observed mainly localized in the cell nucleus, contrasting the localization of MdmX RING domain in the cytoplasm. Mdm2 RING was found to possess an endogenous E3 ligase activity, whereas MdmX RING did not. Both Mdm2 and MdmX RING domains were able to dimerize with endogenous full-length Mdm2 and MdmX protein and affect their cellular function. The results showed that overexpression of the Mdm2 or MdmX RING domains interfered with the endogenous full-length Mdm2 and MdmX activity and resulted in p53 stabilization and p53 target gene activation. However, both Mdm RING domains showed oncogenic activity in a colony formation assay, suggesting that the Mdm RING domains possess p53-independent oncogenic properties. This study highlights the distinct structural and functional traits of the RING domain of Mdm2 and MdmX and characterized their role in cellular responses through interfering with p53 dependent signaling pathway.

## 1. Introduction

The tumor suppressor protein p53, known as the “guardian of the genome”, is dysfunctional in most of human cancers. As a key transcriptional factor, p53 orchestrates cellular responses to DNA damage, nutrient depletion, and abnormal oncogenic events by regulating diverse signaling pathways [1,2,3]. Loss of p53 function causes genomic instability and malignant transformation [4,5,6,7]. In normal cells, p53 is tightly regulated by two MDM (Murine Double Minute) family proteins: Mdm2 and MdmX. Mdm2 and MdmX are homologous in both structure and function, each containing an N-terminal p53 binding domain and a C-terminal RING domain. The two proteins form a dimeric complex through interaction of their RING domains, nevertheless, play non-redundant roles in regulating p53 function [8]. Recent research suggests that Mdm2 primarily inhibits p53 by ubiquitination of p53, which leads to p53 nuclear export and proteasomal degradation [9,10]. MdmX, on the other hand, lacks E3 ligase activity and inhibits p53 through blockade of the transactivation activity of p53 [8,11]. Functional disparity of Mdm2 and MdmX is supported by the mouse knockout studies [12,13,14,15], in which deletion of *MDM2* or *MDMX* resulted in embryonic lethality that could be rescued by concomitant deletion of *TP53*. Phenotypically, *MDM2* knockout showed prevalent apoptosis, whereas *MDMX* knockout caused primarily cell cycle arrest in these genetic studies [11,16,17]. These genetic studies suggest that Mdm2 and MdmX cannot compensate each other and each serves a unique role in the regulation of p53.

Further genetic knock-in studies revealed an interconnected and dependent nature of Mdm2 and MdmX function in vivo. Importantly, deletion of the *MDMX* RING or mutation that impairs Mdm2/MdmX dimerization caused embryonic lethality, which could only be rescued by concomitant *TP53* knockout, despite Mdm2 E3 ligase activity and the ability to interact with p53 were maintained for the mutants [11,18,19]. This study further demonstrates the functional importance of the Mdm2/MdmX heterodimer formation via the RING domain in vivo. Although Mdm2 and MdmX RING domains can interact and form both homo- and heterodimers, MdmX appears to depend on Mdm2 for p53 regulation due to the lack of an NLS signal and intrinsic E3 ligase activity. Through the interaction with Mdm2, MdmX relocates to the nucleus and functions as a negative regulator for p53 transactivation [20]. In vitro studies suggest that MdmX works as a competitive substrate for Mdm2 activity, which results in a more stabilized Mdm2/MdmX ligase complex [19,21,22]. In addition, Mdm2 and MdmX possess p53-independent functions, which contribute to their nonoverlapping physiological roles in the cell. Acting as an E3 ligase, Mdm2 targets a number of cellular proteins for proteasomal degradation, including Foxo3A, pRB, and E-cadherin [23,24,25]. MdmX could interact with mTOR to affect metabolic pathways by impairing mTORC1 activity [26].

Aberrant splice variants of the *MDM2* and *MDMX* genes have been identified from various aggressive forms of cancers. However, the functions of these splice variants remain poorly understood. For instance, MDM2-A and HDM2^ALT1^ are the *MDM2* gene products characterized as lacking the N-terminal p53 binding domain, however, containing the complete C-terminal RING domain [27,28,29,30]. Similarly, the *MDMX* gene splice variant, HDMX211, misses an N-terminal p53-binding domain, but possesses an intact C-terminal RING domain [31]. These splice variants can potentially interact with Mdm2 and MdmX in vivo through dimerization and affect Mdm2/MdmX-dependent suppression of p53 function. However, studies towards understanding the activity and function of these Mdm variants have been inconclusive. In this study, we compared the functions of the Mdm2 and MdmX RING domains and their effects on p53 stabilization and transactivation in a human osteosarcoma U2OS cell line. We show that Mdm2 and MdmX RING domains possess distinct functions in the regulation of the endogenous Mdm2, MdmX, and p53 activity.

## 2. Results

### 2.1. Cellular Localization of the Ectopically Expressed Mdm2 and MdmX RING Domains in U2OS Cells

**Figure 1:**
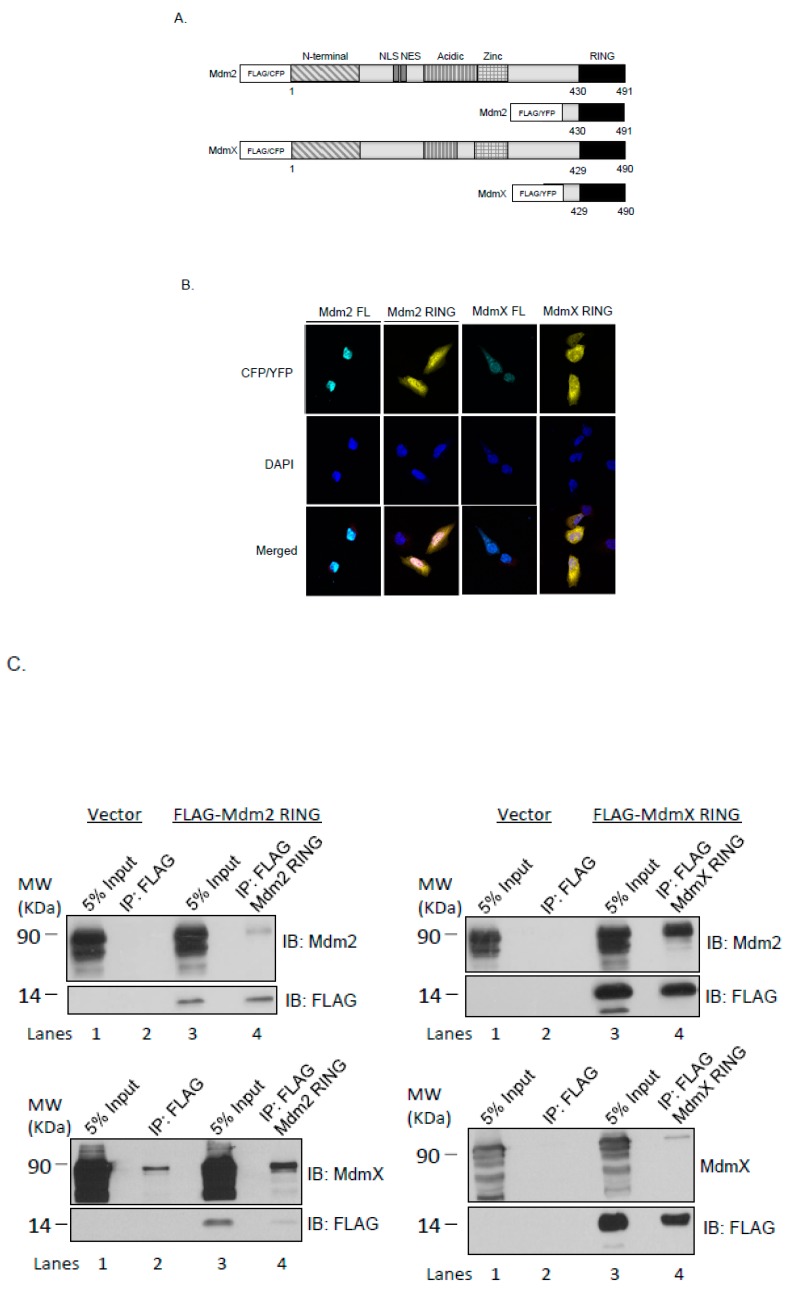
Cellular localization and interaction of the ectopically expressed Mdm2 RING and MdmX RING domains. (**A**) Domain arrangement of Mdm2 and MdmX proteins and constructs used in this study. Both Mdm2 and MdmX possess N-terminal p53-binding domains (N-terminal), central acidic domains (Acidic), zinc finger domains (zinc), and C-terminal RING domains (RING). Mdm2 alone contains a nuclear localization signal (NLS) and a nuclear export sequence. Numbers indicate amino acid residue positions. Wild type full-length Mdm2 and MdmX were expressed as FLAG- or CFP-fusion proteins; Mdm2 and MdmX RING were expressed as FLAG- or YFP-fusion proteins. (**B**) U2OS cells were transfected with CFP-Mdm2, CFP-MdmX, YFP-Mdm2 RING, and YFP-MdmX RING. Cells were fixed at 24 h post-transfection. Nuclei were stained with DAPI. Results indicate nuclear localization of the full-length Mdm2 and overlapping nuclear and cytoplasmic localization of the full-length MdmX, Mdm2 RING, and MdmX RING domains. Scale bars represent 5 μM. (**C**) Co-immunoprecipitation of Mdm2 and MdmX with Mdm2 RING and MdmX RING. U2OS cells were co-transfected with an empty FLAG vector, Mdm2 FL, MdmX FL, FLAG-Mdm2 RING, or FLAG-MdmX RING as indicated to examine the interaction between Mdm RINGs and the full-length Mdm proteins. Co-immunoprecipitation was carried out 24 h post-transfection using a polyclonal anti-FLAG antibody. Immunoblotting was carried out with Mdm2, MdmX and FLAG specific antibodies.

To better understand the functions of Mdm2 and MdmX RING domains, we ectopically expressed the minimal RING domain regions of Mdm2 (Mdm2 RING, residues 417–490) and MdmX (MdmX RING, residues 416–491) as YFP fusion proteins in U2OS cells (Figure 1A). Fluorescence microscopic results showed that Mdm2 RING localized predominantly in the nucleus, while MdmX RING expressed primarily in the cytoplasm, with some weak staining detected in the nucleus (Figure 1B). To compare the subcellular localization of Mdm2 and MdmX RING domain localization with their full-length counterparts, the full-length Mdm2 (Mdm2 FL) and MdmX (MdmX FL) were tagged with CFP and expressed in U2OS cells. Consistent with the previous studies, CFP-Mdm2 FL was found exclusively in the nucleus, while CFP-MdmX FL localized in both the cytoplasm and nucleus (Figure 1B).

### 2.2. Mdm2 RING and MdmX RING Domains Interact with the Full-Length Mdm2 and MdmX

Mdm2 and MdmX RING domains have been identified as the functional domain in several aberrant splice variants of Mdm2 and MdmX identified from cancers, such as HDM2^ALT1^ and HDMX211 [27,31,32]. These variants are expected to affect the functions of endogenous Mdm2 and MdmX by interactions via their RING domains. To examine the interactions of the full-length Mdm2 and MdmX with the Mdm2 and MdmX RING domains in the cell, co-immunoprecipitation experiments were conducted using the U2OS cell line. U2OS cells were co-transfected with full-length Mdm2 or MdmX and FLAG-tagged Mdm2 or MdmX RING domains. As shown in Figure 1C, FLAG-tagged Mdm2 RING domain could readily pull down both Mdm2 FL and MdmX FL. Similarly, FLAG-tagged MdmX RING domain immunoprecipitated both Mdm2 FL and MdmX FL. These results supported the notion that Mdm2 RING and MdmX RING could form dimers with Mdm2 FL and MdmX FL via their C-terminal RING domains. Interestingly, we observed that more MdmX FL was immunoprecipitated by Mdm2 RING domain and more Mdm2 FL was immunoprecipitated by MdmX RING domain, implying that both Mdm2 and MdmX RING domains favor formation of heterodimers.

### 2.3. Ectopically Expressed Mdm2 and MdmX RING Domains Affect the Stability and Function of Endogenous Mdm2 and MdmX

**Figure 2:**
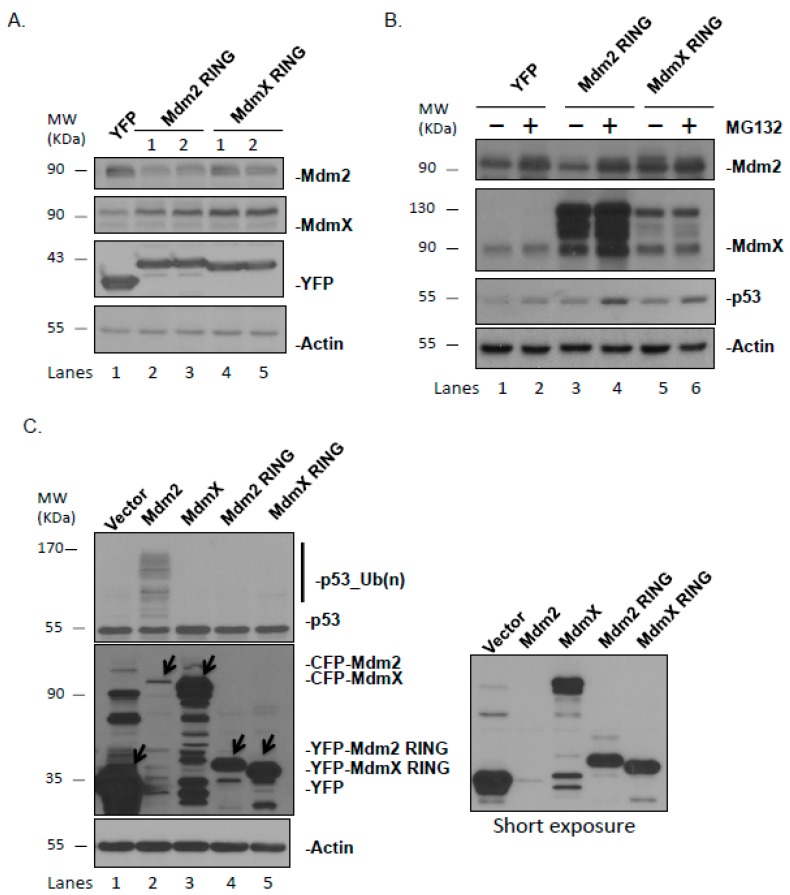
Effects of the ectopically expressed Mdm RING domains on p53 ubiquitination and endogenous levels of Mdm2 and MdmX. (**A**) U2OS cells were transfected with YFP-Mdm2 RING, and YFP-MdmX RING. The total cell lysate was immunoblotted for the protein levels of full-length Mdm2 and MdmX. (**B**) U2OS cells were transfected with YFP-Mdm2 RING and YFP-MdmX RING. The total cell lysate was immunoblotted for the protein levels of full-length Mdm2, MdmX and p53, and compared in the absence and presence of the proteasome inhibitor MG132 treatment. (**C**) U2OS cells were transfected with CFP-Mdm2, CFP-MdmX, YFP-Mdm2 RING, and YFP-MdmX RING. The total cell lysate was immunoblotted for the p53 ubiquitination. The arrows indicate ectopically expressed proteins.

To explore the functions of the isolated Mdm2 and MdmX RING domains, we overexpressed Mdm2 or MdmX RING, and examined their individual effect on the endogenous Mdm2 and MdmX proteins. Overexpression of Mdm2 RING decreased the cellular level of Mdm2 (Figure 2A, middle two lanes). Treatment with MG132, a proteasome inhibitor, restored the level of endogenous Mdm2 in Mdm2 RING overexpressing cells, suggesting Mdm2 RING domain could destabilize the endogenous Mdm2 protein via proteasomal degradation (Figure 2B and Figure S1). Interestingly, Mdm2 RING domain overexpression elevated the endogenous MdmX level and treatment with MG132 further stabilized MdmX, suggesting that the Mdm2 RING domain could cause MdmX proteasomal degradation, but less active than the full-length Mdm2. In the normal cells, endogenous MdmX is dimerized with and degraded by Mdm2. The overexpressed Mdm2 RING could interact with endogenous MdmX and sequester it away from the endogenous Mdm2-mediated degradation. Overexpression of MdmX RING domain stabilized both endogenous Mdm2 and MdmX, whereas MG132 treatment had no effect on levels of endogenous Mdm2 and MdmX (Figure 2A,B). This agrees with inactivity of the MdmX RING as an E3 ligase, and supports its role as a dominant negative of endogenous Mdm2 and MdmX. Its interaction with endogenous Mdm2 and MdmX blocks Mdm2 and MdmX degradation. Lastly, we examined the ability of Mdm2 and MdmX RING domains to ubiquitinate endogenous p53 (Figure 2C). As expected, only the full-length Mdm2 showed activity in polyubiquitination of p53, whereas Mdm2 RING and MdmX RING domains lacking p53-binding sites could not ubiquitinate p53.

### 2.4. Effect of the Ectopic Mdm2 and MdmX RING Domains on the Cellular Level of p53 under Normal and DNA Damage Conditions

As both Mdm2 and MdmX are actively involved in the DNA repair-signaling pathway through p53, the isolated Mdm2 and MdmX RING domains are expected to affect p53 signaling through the interactions with endogenous Mdm2 and MdmX. To study functions of the Mdm2 and MdmX RING domains in the p53 response pathway, FLAG-tagged Mdm2 and MdmX RING were ectopically expressed in U2OS cells, and full-length FLAG-tagged Mdm2 and MdmX were included as positive controls (Figure S2). The effect of the ectopically expressed Mdm2 and MdmX RING on the levels of endogenous Mdm2 and MdmX were tested under DNA damage induced by doxorubicin, an inhibitor of DNA topoisomerase II. Exogenous protein expression was determined with immunoblotting using antibodies against a FLAG-tag. Both FLAG-Mdm2 FL and RING were expressed at low levels compared to MdmX, suggesting that FLAG-Mdm2 FL and RING contained the active RING domain and were less stable (Figure S2).

**Figure 3:**
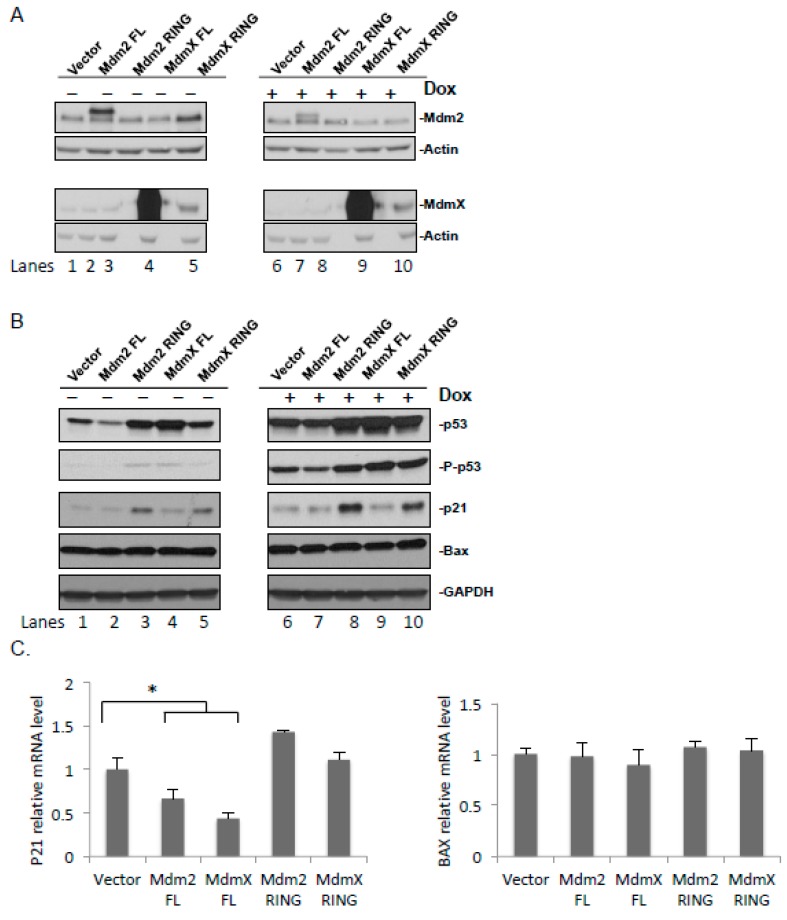
Effects of the ectopically expressed Mdm RING domains on p53 activation. (**A**) U2OS cells were transfected with FLAG-Mdm2, FLAG-MdmX, FLAG-Mdm2 RING, and FLAG-MdmX RING. The total cell lysate was immunoblotted for the protein levels of full-length Mdm2 and MdmX. (**B**) U2OS cells were transfected with FLAG-Mdm2, FLAG-MdmX, FLAG-Mdm2 RING, and YFP-MdmX RING. The total cell lysate was immunoblotted for the protein levels of p53, phosphorylated p53 at Ser15, p21, and Bax. (**C**) U2OS cells were transfected with FLAG-Mdm2, FLAG-MdmX, FLAG-Mdm2 RING, and FLAG-MdmX RING. Relative mRNA expression levels of p21 (*CDKN1A*) and Bax (*BAX*) were analyzed. Results are expressed as Mean ± SEM of three biological replicates. Expression levels of p21 and Bax in the vector control are taken as 1. The results were analyzed using statistical one-way ANOVA test. * *p* ≤ 0.05.

Consistent with the results obtained using YFP-fusion proteins, the MdmX RING domain stabilized both endogenous Mdm2 and MdmX. However, the effect of the Mdm2 RING domain on endogenous Mdm2 and MdmX was not obvious (Figure 3A). The discrepancy might be explained by the relative stoichiometric ratios of the ectopically expressed Mdm2 RING and endogenous Mdm proteins.

The effect of ectopic expression of the Mdm2 and MdmX FL and RING on the level of total p53 as well as the activated p53 (P-p53, phosphorylated at Ser 15) was examined next. In the absence of DOX, both total and activated p53 levels were stabilized in the presence of the ectopically expressed Mdm2 RING and MdmX FL, whereas MdmX RING stabilized p53 to a lesser extent (Figure 3B). A visible decrease was seen in p53 levels following the overexpression of Mdm2 FL. Following DOX treatment, both total and activated p53 levels were stabilized, and more stabilization was observed in the cells ectopically expressing Mdm2 RING, MdmX RING, and MdmX FL (Figure 3B). Overexpression of Mdm2 resulted in decreased steady levels of p53 and phosphorylated p53, agreeing with the role of Mdm2 in p53 ubiquitination and degradation. Overexpression of MdmX FL greatly increased the levels of p53 and phosphorylated p53. It is likely due to the fact that MdmX inhibits p53 transcriptional activity leading to the reduced expression of p53 target genes including Mdm2, while decreased Mdm2 levels further stabilize p53. Consistent with this notion, we observed reduced levels of Mdm2 upon MdmX FL overexpression (Figure 3A, lanes 4 and 9).

### 2.5. Effect of the Ectopic Mdm2 and MdmX RING Domains on p53 Target Genes

External stress stimuli, such as DNA damage, stabilize and activate p53, which in turn regulate transcription of its target genes. To further elucidate the effects of the ectopically expressed Mdm2 and MdmX RING domains on the transcriptional activity of p53, the expression of its downstream target genes, p53-mediated expression of p21 involved in regulation of the cell cycle progression and a proapoptotic Bax protein was investigated (Figure 3B,C) [33,34]. Ectopic expression of Mdm2 RING led to highly increased p21 levels in the presence and absence of DOX. MdmX RING also caused p21 increase, but to a lesser extent compared to Mdm2 RING. The enhancement was considerably greater in the DOX treated samples likely due to the greater amount of activated p53 detected after treatment. In both doxorubicin-untreated and -treated cells, overexpression of Mdm2 FL or MdmX FL led to decreased p21 protein levels. This is consistent with the role of Mdm2 as p53 E3 ligase and MdmX as a potent inhibitor for p53 transactivation. Similar results were observed for *CDKN1A* mRNA levels, suggesting that p21 regulation occurs at both the transcriptional and the protein level. However, no changes were observed in Bax protein and mRNA levels as a result of overexpression of the exogenous proteins.

### 2.6. Effect of the Ectopic Mdm2 and MdmX RING Domains on U2OS Cell Cycle and Cell Growth

**Figure 4:**
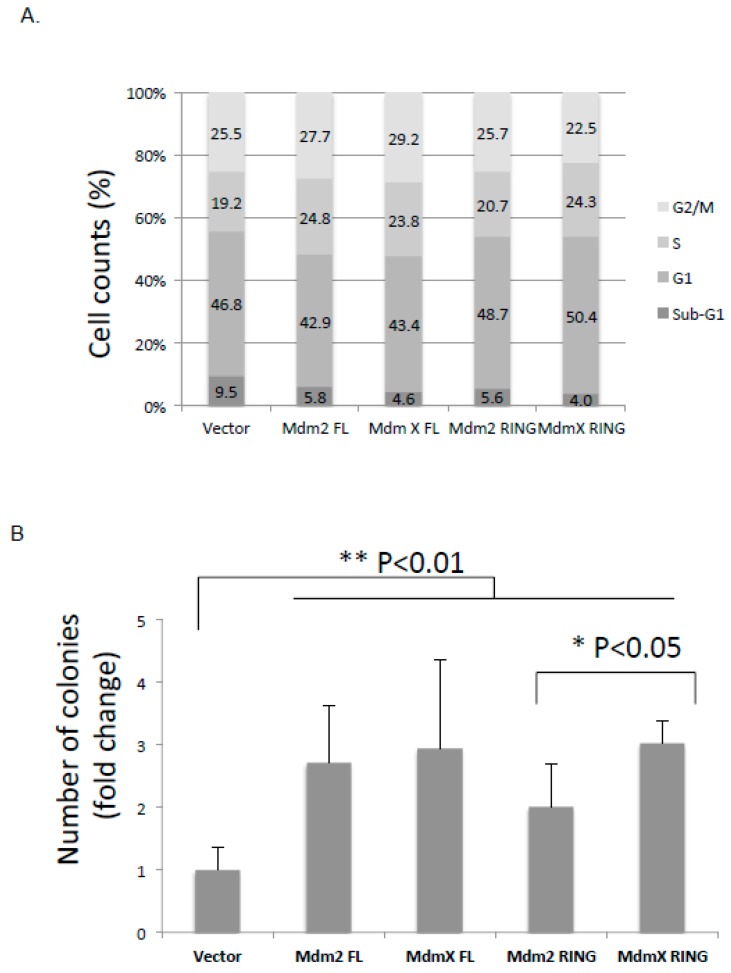
Effects of the ectopically expressed Mdm RING domains on cell cycle and colonogenic activity. (**A**) U2OS cells were transfected with FLAG vector or FLAG tagged-Mdm2, MdmX, Mdm2 RING, or MdmX RING. After a 24-hour transfection, cells were collected, stained with propidium iodide and analyzed for DNA content using flow cytometry. Percentage distribution of cells in each phase of cell cycle (sub-G1, G1, S, and G2/M) was indicated. (**B**) Equal number of U2OS cells were seeded after transfection with FLAG vector or FLAG tagged-Mdm2, MdmX, Mdm2 RING, or MdmX RING. Following a 10-day growing period, cells were washed twice and stained with methylene blue dye. Colonies were counted and normalized using vector as reference. The bar graph represents three independent experiments and results were analyzed using statistical one-way ANOVA test. * *p* ≤ 0.05; ** *p* ≤ 0.01.

To examine the effect of ectopic Mdm2 and MdmX RING domains on U2OS cell cycle progression, flow cytometry analysis was carried out following transfection of U2OS cells with the full-length or RING domains of either Mdm2 or MdmX. Cell DNA contents were stained with propidium iodide and analyzed. We did not observe any significant difference in cell cycle profiles as a result of overexpression of the exogenous proteins. The proportion of cells in G1, S, and G2/M was relatively similar for all the constructs (Figure 4A).

To examine the effect of the ectopic Mdm2 and MdmX RING on U2OS cell growth, clonogenic assays were carried out (Figure 4B). As expected, compared to the vector control group, ectopic overexpression of Mdm2 and MdmX FL showed significantly more colony growth over the 10-day-period (*p* < 0.01). Despite the fact that overexpression of Mdm2 and MdmX RING led to p53 stabilization, ectopic expression of Mdm2 and MdmX RING led to increased colony formation by 2- fold and 3-fold, respectively, compared to the control group. This result suggested that both Mdm2 and MdmX RING promoted tumor growth, but MdmX RING was a more potent tumorigenic construct and comparable to Mdm2 and MdmX FL.

## 3. Discussion

Regulation of p53 by its negative regulators Mdm2 and MdmX is crucial for proper cellular functioning and prevention of tumorigenesis. Mdm2-mediated ubiquitination of p53 remains the main mechanism modulating p53 stability through proteasomal degradation. Mdm2 and MdmX share the most similarity in their RING domains. However, only Mdm2 possesses E3 ligase activity through its RING domain, whereas MdmX carries an inactive RING domain [21,35,36,37]. It is also observed that many human and murine tumors express truncated Mdm2 and MdmX variants, often only containing intact RING domains [27,30,31,32,38]. It is unclear how these Mdm2 and MdmX variants affect the endogenous p53 regulation network or what their specific roles are in tumorigenesis. The purpose of this study was to carry out a comparative analysis of the Mdm2 and MdmX RING domains using human cell lines, focusing on their mechanistic and functional differences in p53 regulation. We first examined the subcellular localization of ectopically expressed Mdm2 and MdmX RING and their interaction with endogenous Mdm2 and MdmX through RING domain. We found that Mdm2 RING localized predominantly in the nucleus and preferentially bound to MdmX. In contrast, MdmX RING resided mostly in the cytoplasm with a small proportion in the nucleus and favored the interaction with Mdm2 through RING domain.

Next, we examine the differential function of Mdm2 and MdmX RING in the regulation of endogenous Mdm2 and MdmX. We found that Mdm2 RING possessed an active E3 ligase activity, but showed opposite effects on the levels of endogenous Mdm2 and MdmX. On one hand, it promoted proteasome-mediated degradation of endogenous Mdm2 through its active RING domain. On the other hand, it formed a heterodimer with endogenous MdmX, which resulted in protection of MdmX from degradation by endogenous Mdm2 and subsequent MdmX stabilization. The net outcome may be dependent on the relative stoichiometric ratios of ectopically expressed Mdm2 RING domain and the endogenous Mdm proteins, which would influence the relative E3 ligase activity of the endogenous Mdm2/MdmX complex [25]. We also found that MdmX RING had no intrinsic E3 ligase activity. It served as a dominant negative of endogenous Mdm2 and MdmX through dimerization and caused stabilization of both Mdm2 and MdmX.

Nuclear localization of Mdm2 was previously reported, and transport between the nucleus and the cytoplasm was enabled due to its nuclear localization signal and nuclear export sequence [39]. MdmX did not possess those motifs, thereby mainly localizing within the cytoplasm [20,40]. However, dimerization with Mdm2 via their C-terminal RING domains could allow transport of MdmX into the nucleus [41]. Nuclear localization of the Mdm2 RING and MdmX RING domains could be explained by a complex formation between the exogenous RING domains and the endogenous Mdm2 that could transport them into the nucleus. Uniform localization of the Mdm2 and MdmX RING domains suggested their ability to regulate Mdm2 and MdmX functions within the nucleus and the cytoplasm.

The effects of the ectopic expression of Mdm2 and MdmX RING on p53 and downstream p53 effects were examined in the absence or presence of DNA damage, induced by doxorubicin. Following a 24-hour transfection in human osteosarcoma cells, we observed the effects of the exogenous proteins on the levels of endogenous p53 and its main negative regulators Mdm2 and MdmX. Furthermore, by means of detecting levels of phosphorylated p53 and its downstream gene targets, namely, p21 and Bax, the gene transcription activity of p53 was examined. The effects of the exogenous proteins were compared between normal and stress conditions, triggered by the DNA-damaging agent doxorubicin.

Following ectopic expression of Mdm2 FL under normal or stress conditions, an increased cellular Mdm2 level and decreased endogenous p53, phosphorylated p53, and p21 levels were found. There were no changes in the endogenous levels of MdmX and Bax. Low p53 levels could be explained by the Mdm2-mediated p53 polyubiquitination, which targeted it for the proteasomal degradation. Consequently, low levels of the phosphorylated p53 and p21 were observed. This was further supported by the fact that Mdm2 FL was primarily localized within the nucleus. In addition, several studies showed Mdm2-dependent destabilization of p21, which could also contribute to the observed low p21 level [42,43].

Overexpression of MdmX FL did not affect levels of endogenous Mdm2 and Bax regardless of doxorubicin administration. Interestingly, under normal conditions, we observed a slight accumulation of p53 and phosphorylated p53, and low levels of p21 expression. Following doxorubicin treatment these effects were not found. Different cell responses to MdmX overexpression indicated functional complexity and differential recruitment of MdmX in normal and stress conditions. An increase in the levels of p53 and phosphorylated p53 could occur as a result of inhibition of the Mdm2-mediated p53 degradation by means of complex formation between Mdm2 and MdmX. Although, p53 was stabilized in the cell, it was transcriptionally inactive as exemplified by the levels of p21 (Figure 3C). This effect was probably seen due to the MdmX-dependent inhibition of the p53 gene transcription activity through interactions between their N-terminal domains. In addition, low p21 level in a sample with the overexpressed MdmX FL could also be explained by several studies that demonstrated MdmX-mediated p21 degradation independent of p53 [44,45]. Note that in contrast to several studies [41], we did not find Mdm2 stabilization by MdmX and efficient p53 degradation by the Mdm2/MdmX heterodimer. The underlying mechanism for this contradiction is not clear; however, it should be addressed in later experiments.

In absence of doxorubicin treatment, ectopically expressed Mdm2 RING domain led to a slight stabilization of endogenous Mdm2 and no changes in MdmX, p53, phosphorylated p53, p21, and Bax levels. Following drug treatment, more efficient stabilization of endogenous Mdm2, an increase in phosphorylated p53 level, and p21 expression were observed. No significant changes in MdmX, p53, and Bax levels were found in presence of a stress signal. Co-immunoprecipitation experiments demonstrated the ability of the overexpressed Mdm2 RING to interact with endogenous full-length Mdm2 and MdmX. Dimerization with Mdm2 could explain nuclear translocation of the exogenous Mdm2 RING from the cytoplasm. Therefore, the observed Mdm2 stabilization in stress conditions could be explained by the dimer formation between endogenous Mdm2 and exogenous Mdm2 RING domain, which led to inhibition of Mdm2 autoubiquitination activity and its degradation. The cellular response to Mdm2 RING overexpression revealed that Mdm2 RING acted in a dominant negative fashion to suppress endogenous Mdm2 E3 ligase function and specifically in presence of a DNA-damaging signal prevented Mdm2-mediated inhibition of the p53 gene transcription activity.

Under normal and stress conditions, stabilization of endogenous Mdm2 and MdmX as a result of MdmX RING overexpression was observed, whereas there was no effect on p53, phosphorylated p53, and Bax levels. The MdmX RING domain was shown to interact with endogenous full-length Mdm2 and MdmX. Stabilization of endogenous Mdm2 and MdmX could be a consequence of inhibition of the Mdm2 E3 ligase activity by means of complex formation between the ectopically expressed MdmX RING and endogenous Mdm2. These findings indicated that the exogenous MdmX RING domain, like Mdm2 RING, exhibited a dominant negative phenotype inhibiting Mdm2 ubiquitination activity, which in turn resulted in p53 stabilization.

Note that despite the stabilization effect of p53 by overexpression of Mdm2 and MdmX RINGs, they enhanced the clonogenicity of tumor cells in a colony-forming assay. The molecular basis for p53-independent tumor-promoting activity of the Mdm2/X RING domains has not been well studied. However, increasing number of studies revealed p53-independent roles of Mdm2 and MdmX in tumor cells. Indeed, Mdm2 RING possesses nucleotide-binding activity [46]. Acting as a chromatin modifier, Mdm2 could contribute to tumorigenesis through attenuating DNA damage response, repressing cell differentiation and impairing amino acid metabolism and redox homeostasis [47,48,49]. Similarly, MdmX could promote genomic instability by association with Nbs1 of the Mre11-Rad50-Nbs1 (MRN) DNA repair complex and inhibition of the DNA damage response in p53- and Mdm2-independent manner [50]. Thus, Mdm2 and MdmX may contribute to tumorigenesis through their RING domain alone.

**Figure 5:**
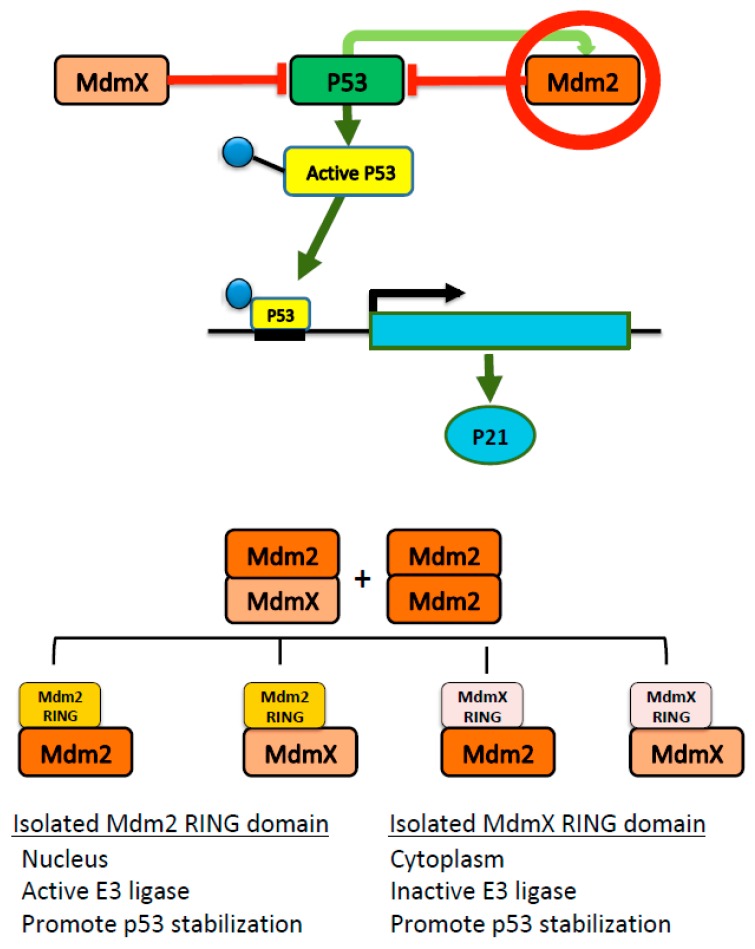
Cartoon scheme of p53-Mdm2-MdmX interplay. Aberrant overexpression of Mdm2 RING domain may dimerize with the full-length Mdm2 or MdmX resulting in inhibition of Mdm2 E3 ligase activity, MdmX stabilization and impaired p53 transcription.

Collectively, this study implies that Mdm2 RING and MdmX RING function differently but inter-connectively in cells depending on the presence or absence of stress stimuli (Figure 5). As expected, Mdm2 RING and MdmX RING could not ubiquitinate p53. However, they served as inhibitors of the endogenous Mdm2 E3 ligase activity, interfering with the p53/Mdm2/MdmX interaction network. Mdm2 RING appeared to be active in mediating Mdm2 degradation through ubiquitin proteolytic system while MdmX RING, lacking E3 ligase activity, stabilized endogenous Mdm2 and MdmX through dimerization. The comparison of the Mdm2 and MdmX RING domains in this study provides functional insight into the role of these RING domains, which is crucial for developing anticancer therapies targeting Mdm2 and MdmX. Note that these findings are based on the overexpression studies using transient transfection, which contain some limitations. Although transient transfection is widely used to study short-term effects in cellular functions [27,51,52], the significance of the long-term effects of Mdm2 and MdmX RING domain on endogenous p53/Mdm2:MdmX remain to be tested in vivo.

## 4. Materials and Methods

### 4.1. Plasmids

Human full-length Mdm2, full-length MdmX, Mdm2 RING (residues 417–491), and MdmX RING (residues 416–490) domains were cloned into pcDNA3.1/YFP, pcDNA3.1/CFP, and pc-DNA3.1/FLAG expression vectors using a standard PCR subcloning technique.

### 4.2. Cell Culture and Transfection

Human osteosarcoma U2OS cells were grown in McCoy’s medium supplemented with 10% FBS. Cells were transfected with plasmids (10 µg of DNA per 10 cm tissue culture plate) using PolyJet transfection reagent (SignaGen Laboratories, Rockville, MD, USA).

### 4.3. Antibodies

The antibodies for immunoblotting and co-immunoprecipitation were mouse monoclonal against Mdm2 (Santa Cruz SMP14, sc-965), MdmX (Millipore 8C6, 04-1555), p53 (Santa Cruz DO-1, sc-126), actin (Santa Cruz C-2, sc-8432), GAPDH (Santa Cruz 0411, sc-47724), YFP-tag (Abm G163), FLAG-tag (Sigma F3165), rabbit monoclonal against phosphorylated p53 (serine 15) (Cell Signaling #9284), p21 (Santa Cruz C-19, sc-397), Bax (Cell Signaling #2772), and rabbit polyclonal against FLAG-tag (Bethyl Laboratories A190-102A). To detect proteins of interest HRP-conjugated anti-mouse IgG (Jackson ImmunoResearch 115-035-166) and anti-rabbit IgG (Jackson ImmunoResearch 111-035-003) antibodies were used.

### 4.4. Immunoblotting

U2OS cells were transfected with YFP (empty vector), CFP-Mdm2 FL, CFP-MdmX FL, YFP-Mdm2 RING, and YFP-MdmX RING or FLAG (empty vector), FLAG-Mdm2 FL, FLAG-MdmX FL, FLAG-Mdm2 RING, and FLAG-MdmX RING. Cells were harvested 24 h post-transfection and lysed in RIPA buffer (50 mM Tris-HCl pH 8.0, 150 mM NaCl, 0.5% Nonidet P-40, 1X protease inhibitor cocktail (Roche, Mannheim, Germany)) followed by 2 sec sonication time with 10% amplitude. Supernatants were boiled in SDS sample buffer for 5 min at 95 °C. Samples were resolved on 12% or 7.5% SDS-PAGE, and immunoblotting was performed using antibodies described above.

### 4.5. Fluorescent Microscopy

U2OS cells were transfected with CFP-Mdm2 FL, CFP-MdmX FL, YFP-Mdm2 RING, and YFP-MdmX RING and grown on coverslips. The 24-hour post-transfection cells were fixed in 1% paraformaldehyde in PBS and permeabilized with 0.5% Triton-X in PBS. Cells were washed with PBS two times. Images were obtained using Zeiss LSM 700 Laser Scanning microscope and LSM software ZEN 2010 (Zeiss, Germany).

### 4.6. Co-Immunoprecipitation

U2OS cells were transfected with CFP-Mdm2 FL and either FLAG-Mdm2 RING or FLAG-MdmX RING, CFP-MdmX FL, and either FLAG-Mdm2 RING or FLAG-MdmX RING to detect protein–protein interactions. Cells were harvested 24 hours post-transfection and lysed in RIPA buffer (50 mM Tris-HCl pH 8.0, 150 mM NaCl, 0.5% Nonidet P-40, 1X protease inhibitor cocktail) followed by 2 sec sonication time with 10% amplitude. Cell lysates were incubated with a rabbit polyclonal anti-FLAG tag primary antibody for 2 hours followed by addition of pre-cleared protein A/G Plus-Agarose beads (Santa Cruz, sc-2003) for 1 hour. Immunoprecipitates were washed with RIPA buffer three times and then boiled in SDS sample buffer for 5 min at 95 °C to release protein complexes from the beads. Samples were resolved on 12% SDS-PAGE, and immunoblotting was performed using antibodies described above.

### 4.7. RNA Expression Analysis

U2OS cells were transfected with pcDNA3.1/FLAG empty vector, FLAG-Mdm2 FL, FLAG-Mdm2 RING, FLAG-MdmX FL, and FLAG-MdmX RING. Cells were harvested after 24- hour transfection and total RNA isolated with Trizol (ThermoFisher, Canada). cDNA was generated using Invitrogen SuperScript Reverse Transcriptase kit. SYBR green kit (SSoFast EveGreen Supermix) from Biorad (Hercules, CA) was used for qPCR. Relative mRNA expression levels were determined using ΔΔCt method. The following oligonucleotides were used:

*CDKN1A* (5’TGAGCGATGGAACTTCGACT3’ and 5’TTAGGGCTTCCTCTTGGAGA3’), 

*BAX* (5’AGACAGGGGCCCTTTTGCTTC3’and 5’TGCAGCTCCATGTTACTGTCC3’).

*GAPDH* (5’AAGGTCATCCCTGAGCTGAAC3’ and 5’ACGCCTGCTTCACCACCTTCT3’).

### 4.8. Cell Cycle Analysis

U2OS cells were transfected with pcDNA3.1/FLAG empty vector, FLAG-Mdm2 FL, FLAG-Mdm2 RING, FLAG-MdmX FL, and FLAG-MdmX RING. Following a 24-hour transfection, cells were fixed in 70% ethanol. After fixing, cells were washed with a staining buffer (0.2% Triton X-100, 1 mM EDTA in 1X PBS). Cells were mixed with staining buffer and RNAseA (100 µg/mL). Cells were stained by propidium iodide (Sigma Aldrich, P4864) for 1 hour. Samples were analyzed in a BD FACScalibur. Analysis was performed in CellQuest Pro (BD Biosciences, San Jose, CA, USA).

### 4.9. Clonogenic Assay

U2OS cells were transfected with pcDNA3.1/FLAG empty vector, FLAG-Mdm2 FL, FLAG-Mdm2 RING, FLAG-MdmX FL, and FLAG-MdmX RING. Following a 24-h transfection, 800 viable cells were seeded in 3-cm cell culture dishes in triplicate for each transfection event. After 10 days, the cells were fixed and stained by methylene blue dye (0.25% methylene blue, 50% methanol). Colonies larger than 1 mm were counted. All the experiments were done in triplicate in each group and repeated. Statistical analyses were performed by using Sigma Stat software. All of the data was represented as Mean ± SEM. Student’s *t*-test was used to compare the values between two groups and one-way analysis of variance (ANOVA), was used to compare the multiple groups. *p* < 0.05 was considered statistically significant.

## Supplementary Materials

Supplementary materials can be found at https://www.mdpi.com/1422-0067/21/4/1309/s1.
