# Supplementary material for: Mdm2 and MdmX RING Domains Play Distinct Roles in the Regulation of p53 Responses: A Comparative Study of Mdm2 and MdmX RING Domains in U2OS Cells
Source: Int J Mol Sci. 2020 Feb 15;21(4):1309. doi: 10.3390/ijms21041309 (PMC7072982; doi:10.3390/ijms21041309)
Supplement: Supplementary file 1 [file ijms-21-01309-s001.pdf]

Figure S1

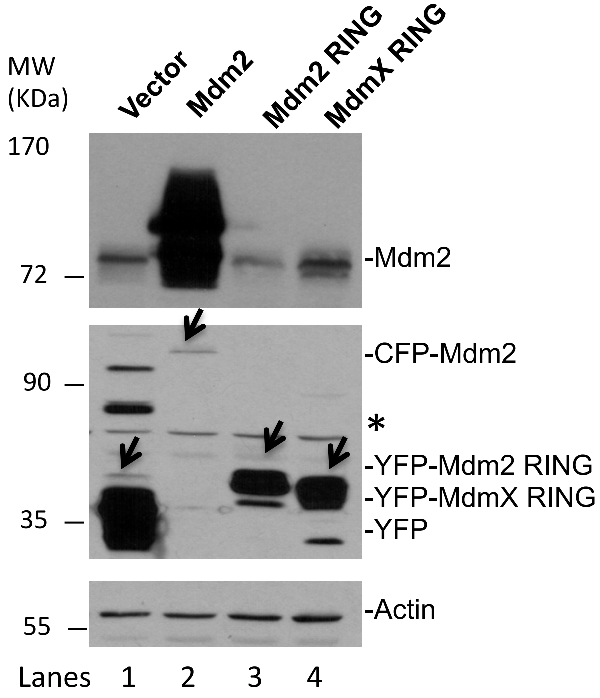

Figure S1. Effects of ectopically expressed YFP-Mdm RING domains on endogenous levels of Mdm2. U2OS cells were transfected with either YFP vector, or one of the CFP/YFP-tagged Mdm protein constructs. Total cell lysate was immunoblotted for Mdm2 levels using antibody SMP14 as well as CFP or YFP expression. Lane 1, U2OS cells with YFP vector (vector); Lane 2, U2OS cells with CFP-Mdm2 full length (Mdm2); Lane 3, U2OS cells with YFP-Mdm2 RING (Mdm2 RING), and Lane 4, U2OS cells with YFP-MdmX RING. Actin was used as loading control. * is noted for the actin bands, since the membrane was re-blotted on the actin blot. Arrows indicate ectopically expressed proteins.

Figure S2

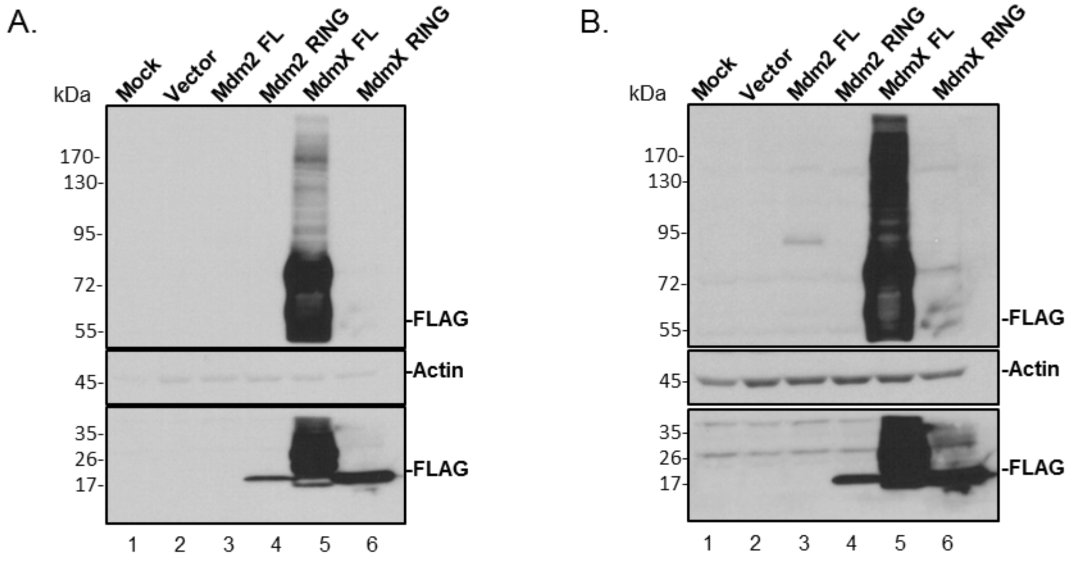

Figure S2. Expression level of the full-length FLAG-Mdm2, FLAG-MdmX, FLAG-Mdm2 RING, and FLAG-MdmX RING for experiments shown in Fig 3A &B. Wild-type full-length Mdm2, MdmX, Mdm2 RING, and MdmX RING domains were expressed as FLAG-fusion proteins. U2OS cells were transfected with an empty FLAG vector, FLAG-Mdm2, FLAG-MdmX, FLAG-Mdm2 RING, and FLAG-MdmX RING. *Mock* sample was prepared by treating cells with the transfection reagent alone with no DNA. Protein samples were prepared 24 hours post-transfection and treated with 500 ng/ml of doxorubicin for 4 hours. Immunoblotting was carried out with a monoclonal anti-FLAG antibody (1:1000). A monoclonal anti-actin (1:1000) antibody was used to track actin. *A,* shorter exposure. *B,* longer exposure.
